# Curcumin Attenuates Both Acute and Chronic Immune Nephritis

**DOI:** 10.3390/ijms21051745

**Published:** 2020-03-04

**Authors:** Tianfu Wu, Bindiya Marakkath, Yujin Ye, Elhaum Khobahy, Mei Yan, Jack Hutcheson, Jiankun Zhu, Xinjin Zhou, Chandra Mohan

**Affiliations:** 1 Department of Biomedical Engineering, University of Houston, Houston, TX 77204, USA; beindia09@gmail.com; 2 Department of Internal Medicine/Rheumatology, University of Texas, Southwestern Medical center, Dallas, TX 75390, USA; graceyeyj@hotmail.com (Y.Y.); EKhobahy@gmail.com (E.K.); mei.yan@utsouthwestern.edu (M.Y.); hutcheson.jack@gmail.com (J.H.); jiankun.zhu@utsouthwestern.edu (J.Z.); 3 Department of Pathology, Baylor University Medical center at Dallas, Dallas, TX 75246, USA; jzhou@pbmlabs.com

**Keywords:** curcumin, immunomodulation, signaling pathways, lupus nephritis, Anti-GBM, mouse models

## Abstract

Curcumin is known to have immunomodulatory potential in addition to anti-oxidant, anti-inflammatory and anti-carcinogenic effects. The aim of the present study is to investigate the therapeutic effects of curcumin on immune-mediated renal disease in an anti-glomerular basement membrane (GBM) model (representing acute kidney Injury, AKI) and murine lupus model (representing chronic kidney disease, CKD). In the AKI model, female anti-GBM 129/svj mice were administered with curcumin right before disease induction. In the CKD model, female MRL.*lpr* mice at the age of 8-10 weeks old were treated with curcumin or placebo via oral gavage daily for two months. After treatment, serum autoantibody levels, splenomegaly and spleen cellularity were reduced in murine lupus. Collectively, curcumin ameliorated kidney disease in the two mouse models with either acute or chronic nephritis, as marked by reduced proteinuria, blood urea nitrogen, glomerulonephritis, crescent formation, tubule-interstitial disease, and renal infiltration by lymphocytes. In addition, curcumin treatment reduced activation of the NFkB, MAPK, AKT and pBAD pathways either systemically, or within the inflamed kidneys. These findings suggest that natural food supplements could become an alternative approach to ameliorating immune-mediated kidney diseases.

## 1. Introduction

Immune-mediated nephritis include lupus nephritis (LN), anti-neutrophilic cytoplasmic antibodies (ANCA)-positive glomerulonephritis, IgA nephropathy (IgAN), membranous nephropathy (MN), and minimal change nephropathy (MCN); they might have common etiologic factors, and eventually could lead to severe persistent proteinuria, chronic renal failure and end- stage renal diseases [1,2,3,4,5,6]. The current treatment for LN involves the use of high-dose glucocorticoids and immunosuppressant, but these are suboptimal and associated with considerable side-effects [7,8,9]. This warrants search for new treatment modalities with improved efficacy and reduced side effects from generalized immunosuppression and drug toxicity. Given a substantial body of research on the therapeutic effects of curcumin, the natural yellow pigment in turmeric isolated from the rhizome of the traditional medicinal plant Curcuma longa [10,11,12,13,14,15,16,17], we speculated that curcumin could ameliorate immune-mediated nephritis.

Recent studies have suggested that curcumin has anti-oxidant, anti-inflammatory and anti-carcinogenic effects and could scavenge free radicals by redox activity [11,12] and enhance cellular antioxidant status by activating Nrf2 transcription factor, a cytoprotective gene [18,19,20,21]. The anti-inflammatory activity of curcumin inhibits the activation of NF-κB, c-Jun and JNK, thereby reducing the levels of proinflammatory cytokines such as TNF-α, IL-1β, and IL-6 [22,23,24]. This inhibition of proinflammatory cytokine secretion by curcumin has been found to reduce adhesion molecules in vitro [25,26]. Inflammation related key enzymes such as prostaglandin E2 synthase, 5-lipoxygenase (5-LO), and cyclooxygenase (COX) could also be inhibited by curcumin. Curcumin inhibits xanthine dehydrogenase and inducible nitric oxide synthase (iNOS) associated with inflammation [27,28] and oxidative stress [29]. Curcumin protects against hepatic and renal injury mediated by inducible nitric oxide synthase during selenium-induced toxicity in Wistar rats [30]. Curcumin abrogates LPS-induced pro-inflammatory cytokine production by macrophages [25]. Randomized, placebo-controlled, double-blind clinical trials have been initiated over the past few years, including in ulcerative colitis [26], Lichen Planus [31], Alzheimer disease [32], type 2 diabetes mellitus [33,34], postoperative fatigue [35] and multiple myeloma [36]. Given the fact that specific signaling pathways including NF-κB were upregulated and hyper-activated in murine lupus B cells [37], and curcumin is able to suppress NF-κB and several other proinflammatory signaling axes, we hypothesize that curcumin could potentially ameliorate disease in mouse models with immune mediated nephritis. In this study, we examine the impact of curcumin on acute nephritis as well as chronic nephritis.

## 2. Results

**Figure 1:**
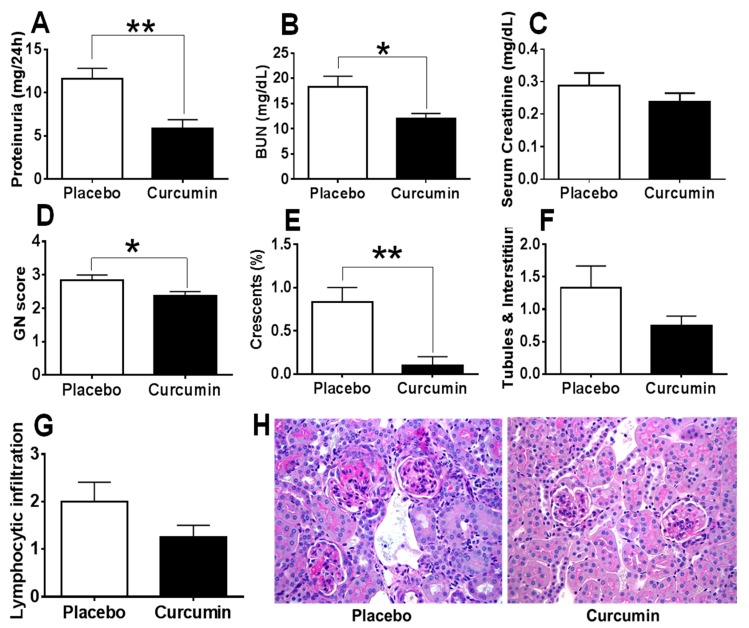
Curcumin ameliorate proteinuria, Blood Urea Nitrogen (BUN) levels and renal pathology in the anti-glomerular basement membrane (GBM) mouse model. Upon treatment with curcumin, anti-GBM subjected mice (model for acute immune nephritis) demonstrated reduced 24-h proteinuria (**A**), BUN (**B**), serum creatinine (**C**), GN score (**D**), crescent formation (**E**), tubules and interstitial pathology (**F**) and lymphocytic infiltration (**G**), compared to the placebo group. H&E staining of kidney is also shown in (**H**). Shown are representative photomicrographs of Periodic acid–Schiff (PAS) stained kidney sections isolated from curcumin-treated and placebo treated mice. All images were taken at 200× total magnification. Data were compared using a two tailed Student’s *t*-test. * represents *p* < 0.05, ** represents *p* < 0.01.

Anti-glomerular basement membrane (GBM) antibodies injected to the 129 strain of mice have previously been shown to cause crescentic glomerulonephritis and proteinuria within 2–3 weeks [38,39] and this is used in this study as a model of acute immune nephritis. In contrast, MRL.*lpr* mice, one of the best studied models for spontaneous lupus with autoantibodies and glomerulonephritis similar to human lupus is used as the chronic kidney disease model, also mediated by autoantibodies [38,39]. Curcumin was administered daily from D0 for 15 days. Upon sacrifice of the mice on D15 (peak of disease), 24-h urine and blood were collected for further analysis and kidneys were processed for renal pathology as described elsewhere [37,38,40]. Twenty-four-hour proteinuria, Blood Urea Nitrogen (BUN) and serum creatinine were measured as described previously [37,40]. As shown in Figure 1, clearly the 24-h proteinuria (Figure 1A) and BUN (Figure 1B) were significantly reduced in the curcumin-treated group compared to the placebo group, *p* < 0.05. In addition, serum creatinine levels (Figure 1C) were also decreased in the curcumin-treated mice, but the difference did not reach statistical significance (*p* > 0.05). Importantly, glomerulonephritis (GN) score (Figure 1D), the percentage of crescent formation (Figure 1E), tubules and interstitial score (Figure 1F) and the periglomerular and perivascular lymphocytic infiltration in the kidney (Figure 1G) were remarkably reduced in the curcumin-treated group compared to the placebo group. H&E staining of renal sections demonstrated the improvement of renal pathology after curcumin treatment, as marked by reduced glomerular size and inflammation, and reduced mesangial deposits in the glomeruli (Figure 1H).

**Figure 2:**
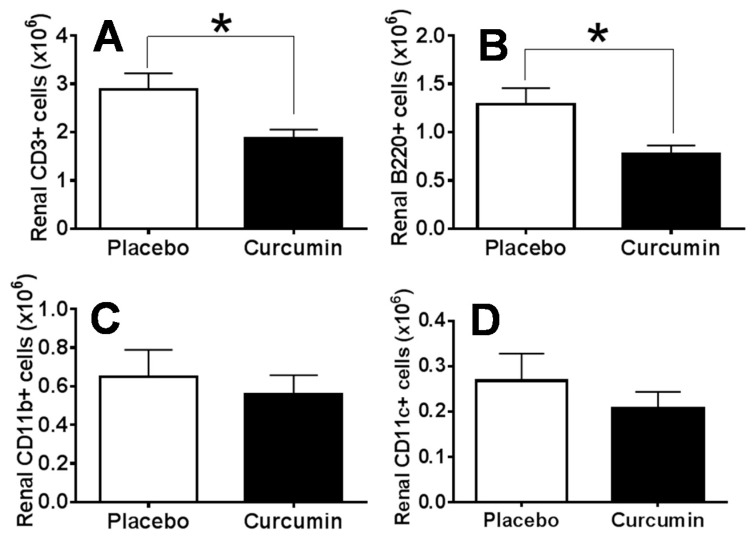
Treatment with curcumin decreased renal lymphoid cell infiltration in anti-GBM mouse model. Single cell suspensions were prepared from the isolated kidney samples and cells were counted on Cellometer AutoM10 automated cell counter (Nexcelom Bioscience, Lawrence, MA). Comparison of the mean values of the total renal cell numbers in the curcumin-treated group and the placebo group by a Student’s t test did not show a significant difference. The mean values of the total number of CD3+ cells (**A**, *p* < 0.05) and B220+ cells (**B**, *p* < 0.05) were significantly reduced in the curcumin-treated group compared to the placebo group. Renal CD11b+ cells and CD11c+ cells were also decreased in the curcumin-treated mice; however, the difference was not statistically significant (**C**,**D**, *p* > 0.05).

In order to determine how curcumin might impact cell subsets and activation status of infiltrating lymphocytes in the kidney of anti-GBM mice, we harvested kidneys to prepare single cell suspension. Cells were then counted using the Cellometer Auto M10 automated cell counter (Nexcelom Bioscience, Lawrence, MA, USA). Single cell suspensions were stained for flow cytometry analysis of the lymphocyte subsets including CD3+ cells, B220+ cells, CD11b+ cells, and CD11c+ cells. Comparison of the mean values of the total renal cell numbers in the curcumin-treated group and the placebo group using the Student’s *t*-test did not show a significant difference. The mean values of the total number of CD3+ cells (Figure 2A) and B220+ cells (Figure 2B) were significantly reduced in the curcumin-treated group compared to the placebo group. Likewise, CD11b+ cells and CD11c+ cells were also reduced in the curcumin-treated group compared to the placebo group in the anti-GBM mouse model, but the difference was not statistically different (Figure 2C,D).

**Figure 3:**
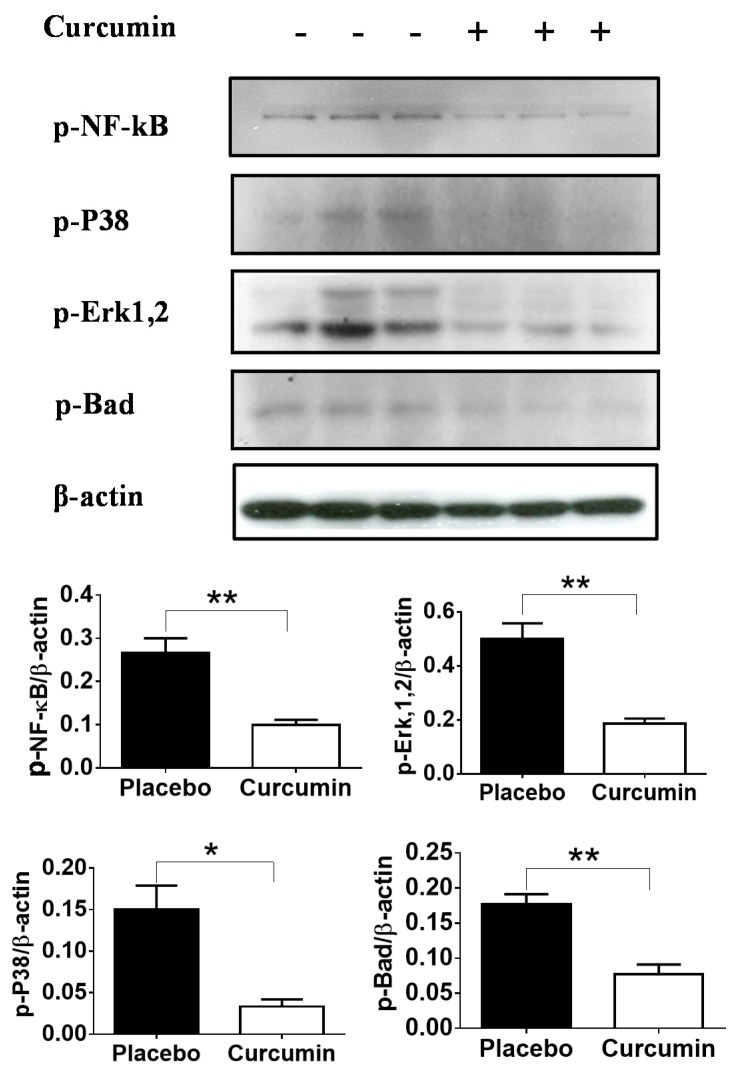
Changes in cell signaling in total renal cortex lysates after curcumin treatment in the anti-GBM mouse model. Western blot analyses showed significantly reduced phosphorylation of NF-κB, P38, Erk1,2 and Bad in renal tissues of mice treated with curcumin compared with the group treated with placebo. Since total renal cortex lysates were used, this is likely to reflect signaling status in both the renal parenchymal cells as well as infiltrating immune cells. The intensity of the Western blot band was further quantified using ImageQuant software (ThermoFisher) and the data were plotted using Prism GraphPad software. Data shown are representative of three mice in each group. Each bar represents the mean/SEM of three mice. Error bars denote standard deviation. * represents *p* < 0.05, ** represents *p* < 0.01.

To determine the impact of curcumin on various cell signaling pathways in kidney, Western blot was performed using total renal cortex lysates prepared from the kidneys of the anti-GBM afflicted mice. Since total renal cortex lysates were used, this is likely to reflect signaling status in both the renal parenchymal cells as well as infiltrating immune cells. The results demonstrated that phosphorylation of NF-κB, p38, extracellular signal-regulated kinases (Erk1,2) and Bad were significantly reduced in the renal tissues of mice treated with curcumin compared with the placebo group (Figure 3). This suggests that curcumin might improve renal pathology by inhibiting multiple signaling pathways, which are responsible for lymphoproliferation and inflammation such as NF-κB, P38 and Erk1,2 or apoptosis-promoting molecules, such as Bad.

**Figure 4:**
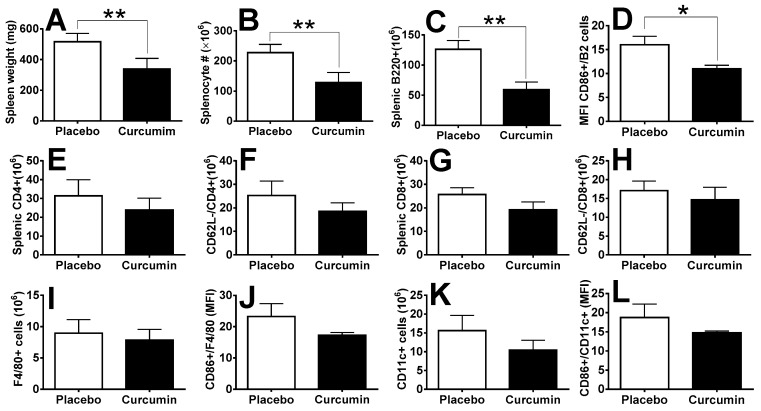
Curcumin ameliorates splenomegaly and lymphocyte numbers and activation in the MRL.*lpr* spontaneous lupus mouse model. Curcumin treatment significantly reduced splenomegaly (**A**) and splenocyte number (**B**) in MRL.*lpr* mice compared to placebo mice. B cell (**C**,**D**), T cell (**E**–**H**), macrophage (**I**,**J**) and dendritic cell (**K**,**L**) populations and their activation status were also impacted by curcumin treatment. * indicates *p* value < 0.05, ** indicates *p* value < 0.01 as compared to the placebo group.

Next, we investigated the preventive effect of curcumin on spontaneous murine lupus using the MRL.*lpr* mouse model at the age of 8~10 weeks, over a course of 2 months. We found both splenomegaly (Figure 4A) and total splenocytes (Figure 4B) were significantly reduced in the curcumin-treated group compared to the placebo group. Splenic B220+ B cells were also significantly reduced after curcumin treatment (Figure 4C), and CD86+ activated B-cells were significantly reduced as well (Figure 4D). We also found both the CD4+ and CD8+ T cell subsets to be decreased after curcumin treatment (Figure 4E,G); likewise, CD4+CD62L− T cells and CD8+CD62L− T cells were also decreased (Figure 4F,H), suggesting the suppressive effect of curcumin on the activation of T cells in MRL.*lpr* mice, although these changes were not statistically significant (*p* > 0.05). Likewise, F4/80+ macrophages (Figure 4I,J) and CD11c+ dendritic cells (Figure 4K,L) were decreased, together with their cell activation status (CD86+) in the curcumin-treated group compared to the placebo group, although these changes did not reach statistical significance in this particular study.

**Figure 5:**
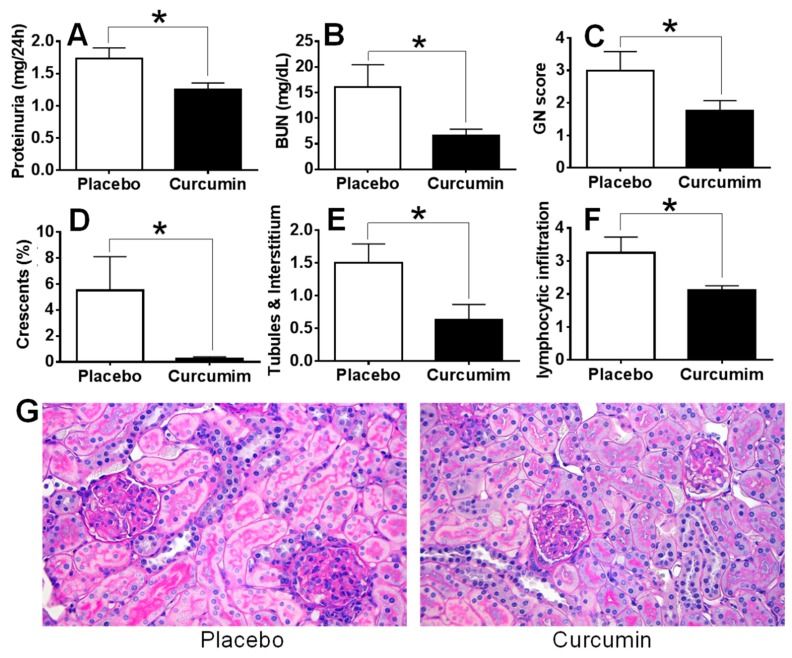
Curcumin treatment suppressed renal disease in MRL.*lpr* mice. After treatment of MRL.*lpr* female mice with curcumin for 2 months, proteinuria (**A**) and serum BUN (**B**) levels were measured. Renal sections were evaluated for glomerulonephritis as evidenced by the GN score (**C**), glomerular crescent percentage (**D**), tubulointerstial disease score (**E**) and periglomerular and perivascular lymphocytic infiltration score (**F**). All these renal disease phenotypes were significantly alleviated in the curcumin-treated group compared to the control group. H&E staining of the paraffin-embedded renal sections from MRL.*lpr* are also shown (**G**) for both curcumin-treated group and placebo group. Clearly, renal disease pathology was improved after curcumin treatment. Images are representative of sections from 5 mice in each group (original magnification 200×). * indicates *p* value < 0.05 as compared to the Placebo group.

We next ask if curcumin is also beneficial in preventing the development of chronic kidney disease-lupus nephritis. After the treatment of MRL.*lpr* female mice (8–10 weeks old) with curcumin at 1 g/kg bodyweight, daily for 2 months, we found that 24-h proteinuria (Figure 5A) and serum BUN (Figure 5B) levels were significantly reduced compared to the placebo group, *p* < 0.05. Renal sections were evaluated by a renal pathologist for glomerulonephritis as evidenced by the GN score (Figure 5C), glomerular crescent percentage (Figure 5D), tubulointerstitial disease score (Figure 5E) and periglomerular and perivascular lymphocytic infiltration score (Figure 5F). All these renal disease manifestations were significantly alleviated in the curcumin-treated group compared to the placebo group, *p* < 0.05. H&E staining of paraffin-embedded renal sections from representative mice is also shown (Figure 5G) for both the curcumin-treated group and the placebo group. Clearly, nephritis-associated kidney pathology was significantly improved after curcumin treatment, as marked by reduced glomerular size and inflammation, reduced mesangial deposits in the glomeruli, and reduced tubular casts.

**Figure 6:**
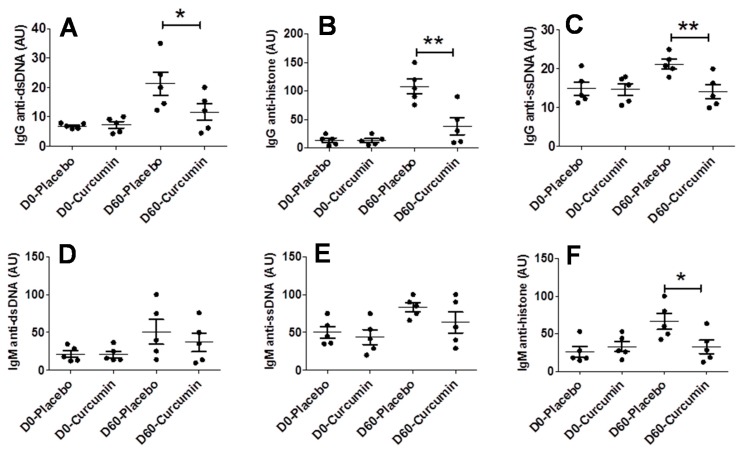
Curcumin dampens autoantibody production in a spontaneous lupus mouse model MRL.*lpr*. Various serum autoantibodies were examined including IgG anti-dsDNA (**A**), IgG anti-ssDNA (**B**), IgG anti-histone (**C**), IgM anti-dsDNA (**D**), IgM anti-ssDNA (**E**), IgM anti-histone (**F**). ELISA was used to detect the indicated autoantibodies in sera isolated from mice treated with either curcumin or placebo on the indicated days of treatment. Each data point represents one mouse. All values are optical density (OD) readings at 405 nm wavelength. Line represents mean ± SEM. Data were compared using a two-tailed Student’s *t*-test. * represents *p* < 0.05, ** represents *p* < 0.01.

We next examined whether curcumin could impact immune system changes in lupus, such as autoantibody production. We collected D0 and D60 sera and measured IgG anti-dsDNA, IgG anti-ssDNA, IgG anti-histone, IgM anti-dsDNA, IgM anti-ssDNA and IgM anti-histone at both the starting point (D0) and end point (D60) of the treatment. All assayed autoantibodies were low in both the curcumin-treated group and placebo group on Day 0, which serve as a baseline for each autoantibody type (Figure 6). Compared to the placebo group, curcumin-treated mice exhibited significantly reduced IgG anti-dsDNA (Figure 6A), IgG anti-ssDNA (Figure 6B), IgG anti-histone (Figure 6C) and IgM anti-histone (Figure 6F) following treatment; however, there was no significant difference in IgM anti-dsDNA (Figure 6D) and IgM anti-ssDNA (Figure 6E) levels after curcumin treatment.

**Figure 7:**
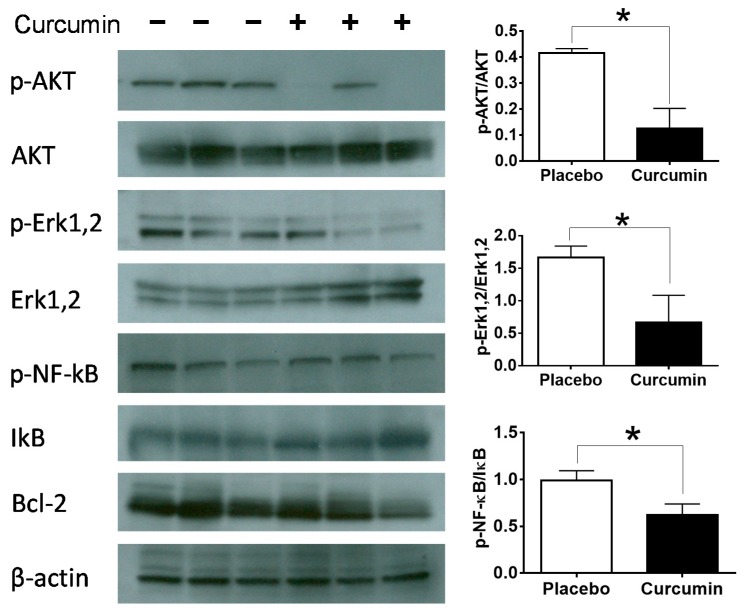
Cell signaling changes caused by curcumin treatment in the splenocytes of MRL.*lpr* mice. Western blot was performed to examine the impact of curcumin on cell signaling of immune cells. The phosphorylated form and/or the pan form of AKT, Erk1,2, NF-κB, IκB and Bcl-2 were examined and the intensity of each band of interest was quantified using ImageQuant and the data were plotted using Prism Graphpad. Data shown are representative of three mice in each group. β-actin antibody was used as a loading control. Each bar represents the mean of three mice and the error bars denote standard deviation. * represents *p* < 0.05.

Next, we examined whether curcumin could impact various cell signaling pathways of immune cells in the spleen. The phosphorylated form and/or the pan form of AKT, Erk1,2, NF-κB, IκB and Bcl-2 were examined and the intensity of each band of interest was quantified using ImageQuant. Compared to the placebo group, we could clearly document significant inhibition of the phosphorylation of AKT, Erk1,2 and NF-κB after curcumin treatment, *p* < 0.05 (Figure 7).

**Figure 8:**
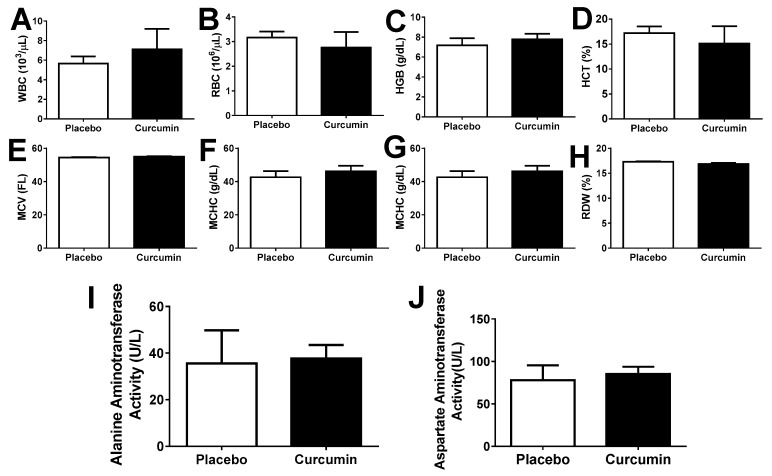
Sixty-day curcumin treatment did not cause overt side effects. Complete Blood Count (CBC) was performed in a clinical laboratory, after a 60-day curcumin and placebo treatment of MRL.*lpr* (female, *n* = 5 each). Peripheral blood cell counts were not altered by curcumin treatment in the immune nephritis mouse models (**A**–**H**). Enzymes for liver function: Alanine amino transferase and aspartate amino transferase enzyme activities were measured in the immune nephritis mouse models (**I**,**J**) to compare the curcumin treatment with similar samples from the placebo treated mice. Each bar represents the mean of three mice. Error bars denote standard deviation.

Finally, we determined whether curcumin treatment could cause any side effects in the immune nephritis mouse models. Complete blood counts (Figure 8A–H) as well as liver function-related enzymes alanine amino transferase and aspartate amino transferase enzyme levels (Figure 8I,J) in blood were examined in curcumin-treated and placebo mice; however, neither blood cell counts nor liver function were negatively affected by curcumin [41].

## 3. Discussion

In addition to the recent evidence from mechanism-based studies on the potential of curcumin in the prevention and treatment of cancer and inflammatory diseases, there has also been some effort to unravel the immunomodulatory effects of curcumin and examine its use in autoimmune diseases like rheumatoid arthritis [42], type-1 diabetes [43], psoriasis [44] and inflammatory bowel disease [45]. Curcumin has modulatory effects on various immune cells via relevant signaling pathways [15]:

(1) In T cells, curcumin inhibited proliferation of lymphocytes derived from fresh spleen induced by tumor promoter phorbol-12-myristate ester-13-acetate (PBA) [46]. Curcumin blocks interleukin (IL)-2 signaling in T-lymphocytes by inhibiting IL-2 synthesis, CD25 expression, and IL-2 receptor signaling [47]. Inhibition of Calcium-ATPase activity and Calcium transport by curcumin prevents dephosphorylation of NFAT and thereby prevents expression of genes for activation of T cells [48]. In this study, we observed both CD4+ and CD8+ T cell subsets were decreased after curcumin treatment (Figure 4E,G); likewise, CD4+CD62L− T cells and CD8+CD62L− T cells were decreased (Figure 4F,H). These results are in agreement with the results from others, confirming the suppressive effect of curcumin on the proliferation and activation of T cells. In a study by Hyojung Lee et al., curcumin was shown to decrease proteinuria, serum levels of antibodies, IgG immune complex in glomeruli and renal inflammation in NZB/W F1 female mice. The protective effect was speculated to be due to the interaction of curcumin with T_reg_ cells as the therapeutic effects disappeared after Treg depletion by anti-CD25 antibody injection [42].

(2) In B cells, curcumin could arrest growth and promote apoptosis of B cell lymphoma by downregulation of various signaling molecules including Early Growth Response Protein 1 (EGR-1), C-myc, Bcl-XL, NF-κB, and p53 [49]. In another study, curcumin was found to induce apoptosis in Chronic lymphocytic leukemia (CLL) B cells in a dose-dependent manner and inhibited constitutively active pro-survival pathways including STAT3, AKT, and NF-κB. The expression of the anti-apoptotic proteins Mcl-1 and X-linked inhibitor of apoptosis protein (XIAP) is suppressed by curcumin. Curcumin also up-regulated the pro-apoptotic protein BIM [50]. In this study, we demonstrated that B220+ B cells were significantly reduced after curcumin treatment (Figure 4C), and that CD86+-activated B cells were significantly reduced as well (Figure 4D). The examination of signaling pathways in splenocytes indicated that cell proliferation-related signaling molecules such as AKT, Erk1,2 and NF-κB were downregulated, which suggests that curcumin may suppress autoimmunity via the amelioration lymphoproliferation. Interestingly, the anti-apoptotic molecule Bcl-2 was also downregulated upon curcumin treatment, which is consistent with the speculation that curcumin may abrogate immune responses via promoting apoptosis of immune cells in the peripheral lymphoid tissue such as the spleen in lupus.

(3) Curcumin could inhibit nitric oxide (NO) production in macrophages [51]. LPS-induced phosphorylation of p65 subunit of NF-κB and IκBα in murine macrophages was blocked by curcumin [52]. Curcumin treatment significantly reduced macrophage recruitment in a mouse model of peritonitis. Monocyte-derived cell lines treated and primary human macrophages treated with curcumin significantly inhibited cell migration in vitro [52,53]. Curcumin was found to impair the T cell stimulatory function of dendritic cells with reduced secretion of proinflammatory cytokines and nitric oxide (NO) and low surface expression of co-stimulatory molecules, leading to an overall diminished antigen-presenting cell activity in accelerated murine models of Type 1 diabetes [54]. Curcumin could induce DC differentiation towards maturation-arrest. DC treated with curcumin (CurcDC) demonstrated minimal CD83 expression, down-regulation of CD80/CD86 and reduction in both major histocompatibility complex (MHC) class II and CD40 expression. CurcDC displayed decreased RelB and interleukin (IL)-12 mRNA and protein expression. Allo-stimulatory capacity of CurcDC was functionally decreased [55]. Interestingly, in our renal cell flow cytometry analysis, we noticed both macrophages (CD11b+) and dendritic cells (CD11c+) to be decreased after the treatment of anti-GBM mice with curcumin, although not attaining statistical significance. These observations are in agreement with previous results by others regarding the inhibitory effects of curcumin on macrophages and dendritic cells. This suggests that curcumin may suppress the proliferation and activation of infiltrating macrophages and dendritic cells in the inflamed kidneys during immune nephritis. Indeed, the significant improvement of renal pathology we observed in both the spontaneous lupus mouse model and in the anti-GBM mouse model support this notion.

Curcumin could be useful in immune nephritis and systemic autoimmunity by modulating multiple pathways. Among these, histone acetylation-induced epigenetic dysregulation has been implicated in the pathogenesis of lupus. Alteration of histone acetylation status through histone deacetylase (HDAC) inhibitors is a newly investigated avenue to target lupus [42]. Of relevance, studies have shown that curcumin inhibits histone acetyl transferase (HAT) inducing histone hypoacetylation in vivo without involving HDAC [42].

Recent studies have shown that curcumin ameliorates autoimmune diseases by regulating inflammatory cytokines such as IL-1beta, IL-6, IL-12, TNF-alpha and IFN-gamma and associated JAK-STAT, AP-1, and NF-κB signaling pathways in immune cells, as reviewed by others [56]. In a psoriasis mouse model, curcumin significantly inhibited secretion of inflammatory factors including interleukin (IL)-17, IL-22, IFN-γ, IL-2, IL-8 and TNF-α in T cells by 30%–60% in vitro. Indeed, inflammatory factors including TNF-α, IFN-γ, IL-2, IL-12, IL-22 and IL-23 in mouse serum were decreased by curcumin treatment in vivo [57]. These observations support our hypothesis that curcumin might suppress inflammation in both acute and chronic immune nephritis via the downregulation of transcription factors such as NF-κB, which dictate the expression of proinflammatory cytokines.

It is encouraging that curcumin use in a randomized and placebo-controlled study of 24 patients with relapsing or refractory biopsy proven LN led to a decrease in proteinuria, hematuria and blood pressure following short-term curcumin treatment [58]. Hence, it is promising to note that curcumin could serve as a natural remedy for the treatment of immune-mediated nephritis, including lupus nephritis. Clearly, larger clinical trials are warranted.

## 4. Materials and Methods

### 4.1. Mice and Treatment

Mouse strains 129/svj and MRL.*lpr* were obtained from the Jackson Laboratory (Bar Harbor, ME, USA). Animal experiments were approved by the Institutional Animal Care and Use Committee (IACUC) at the University of Texas, Southwestern Medical Center, Dallas, Texas (Approved animal protocol # 0841-07-19-1 on 5 January 2010).

For the acute nephritis model, female 129/svj mice (*n* = 10 per group) were pre-sensitized on Day 1 with rabbit IgG (250 μg per mouse, intraperitoneal injection), in adjuvant as described previously [38,39]. On Day 5, the mice were challenged intravenously with nephrotoxic serum or NTS (200 μg per 25 g body weight). For the chronic nephritis model, we treated 8–10 weeks old female MRL.*lpr* mice (*n* = 5 per group) with curcumin (1 g/kg body weight) or placebo (sesame oil) via oral gavage daily for two months. After treatment, proteinuria, serum antibody levels, spleen cellularity, renal pathology and splenic cell signaling status were examined. Curcumin (a kind gift from Dr. Bharat Agarwal at MD Anderson Cancer Center, 1 g/kg body weight) or placebo (sesame oil) via oral gavage was given daily to mice starting on Day 0 for 15 days. Then the mice were sacrificed and proteinuria, renal cellularity, pathology, and cell signaling status were examined.

### 4.2. Renal Disease

Metabolic cages were used for twenty-four-hour urine samples collection. Total urinary protein was assayed using Coomassie-based assay (Thermo Fisher Scientific, Waltham, MA, USA) [38]. Blood samples were collected on Day 15. Blood urea nitrogen (BUN) was assayed using a colorimetric method purchased from Sigma-Aldrich and serum creatinine was assayed using a kit obtained from Caymen Chemical (Ann Arbor, MI, USA). Upon sacrifice of mice, kidneys were fixed, sectioned, and stained with hematoxylin and eosin, and periodic acid schiff. At least 100 glomeruli were studied per section by light microscopy for features of inflammation and/or tissue damage, and graded as detailed elsewhere [40], in a blinded manner. Glomeruli were screened for the presence of hypertrophy, proliferative changes, hyaline deposits, crescent formation, fibrosis/sclerosis, as well as basement membrane thickening. Glomerulonephritis (GN) score or severity was graded on a 0–4 scale as listed here: 0, normal; 1, mild increase in mesangial cellularity and matrix; 2, moderate increase in mesangial cellularity and matrix, with thickening of the GBM; 3, focal endocapillary hypercellularity with obliteration of capillary lumina and a substantial increase in the thickness and irregularity of the GBM; and 4, diffuse endocapillary hypercellularity, segmental necrosis, crescents, and hyalinized end-stage glomeruli. Similarly, the severity of tubulointerstitial nephritis (TIN) was graded on a 0–4 scale, based on the degree of tubular atrophy, inflammatory infiltrates, and interstitial fibrosis, as detailed previously [38,39,40].

### 4.3. Flow Cytometric Analysis

Splenocytes were depleted of erythrocytes using a lysis buffer (with 0.15 M NH_4_Cl, 10 mM KHCO_3_, 0.1 mM Na_2_EDTA, pH 7.2), and single cell suspensions were subjected to flow cytometric analysis, as reported previously [37,40].

### 4.4. ELISA for Autoantibodies

The anti-dsDNA, anti-histone, and anti-histone/DNA ELISA assays were executed out as reported elsewhere [37,40]. All OD values were converted to Units/mL (U/mL), using a positive control serum obtained from MRL.*lpr* mice, by setting the binding by a 1:100 dilution of this serum to equal 100 U/mL. Sera samples with reactivity stronger than the highest standard were further diluted and re-assayed. The glomerular binding assay was executed as reported previously [37,40], using sonicated rat glomeruli as the plate bound substrate.

### 4.5. Western Blot Analysis

Spleen or kidney tissues were lysed with 20 mM Tris-HCL (pH7.5), 150 mM NaCl, 1 mM Na_2_EDTA, 1 μg/mL leupetin, 1% Triton X-100, 1 mM Na_3_VO_4_, and the obtained protein was quantified using the Bradford assay, and 10 μg was ran per lane in SDS-PAGE gels. Primary antibodies Abs against these signaling molecules were purchased from Cell Signaling Technology (Danvers, MA, USA): AKT, phospho-AKT, NF-κB, phospho-NF-κB, phospho-P38, Erk1,2, phospho-Erk1,2, IκB, phospho-Bad; Antibody against Bcl-2 was purchased from Santa Cruz Biotechnology (Dallas, TX, USA). Anti-β-actin served as the loading control (Advanced Immunochemical Inc, Long Beach, CA, USA). HRP-conjugated secondary antibody reagents and an ECL-plus detection assay (GE Healthcare, Chicago, IL, USA) were used to develop the blot. All band intensities were quantified with ImageJ software 1.37 (National Institute of Health, Bethesda, MD, USA, http:rsb.info.nih.gov/ij).

All chemicals were purchased from Sigma-Aldrich (St. Louis, MO, USA) unless otherwise specified above.

### 4.6. Statistics

Statistical tests were performed using a paired and unpaired student’s T test, using Graphpad Instat 3 (GraphPad Software, San Diego, CA, USA). For depicted experiments, the mean and SEM are also shown. A *p* value < 0.05 was considered significant.

## 5. Conclusions

To sum, curcumin therapy ameliorated kidney disease in the two mouse models with either acute or chronic nephritis, as marked by reduced proteinuria, blood urea nitrogen, glomerulonephritis, crescent formation, tubule-interstitial disease, and renal infiltration by lymphocytes. In addition, curcumin treatment reduced activation of the NFkB, MAPK, AKT and pBAD pathways either systemically, or within the inflamed kidneys. These findings suggest that natural food supplements could become an alternative approach to ameliorating immune-mediated kidney diseases.

## References

[1] Nasr S.H., D’Agati V.D., Park H.-R., Sterman P.L., Goyzueta J.D., Dressler R.M., Hazlett S.M., Pursell R.N., Caputo C., Markowitz G.S. (2008). Necrotizing and crescentic lupus nephritis with antineutrophil cytoplasmic antibody seropositivity. Clin. J. Am. Soc. Nephrol..

[2] Couser W.G. (2012). Basic and translational concepts of immune-mediated glomerular diseases. J. Am. Soc. Nephrol..

[3] Weening J.J., D’agati V.D., Schwartz M.M., Seshan S.V., Alpers C.E., Appel G.B., Balow J.E., Bruijn J., Cook T., Ferrario F. (2004). The classification of glomerulonephritis in systemic lupus erythematosus revisited. Kidney Int..

[4] Jennette J.C., Wilkman A.S., Falk R.J. (1989). Anti-neutrophil cytoplasmic autoantibody-associated glomerulonephritis and vasculitis. Am. J. Pathol..

[5] Donadio J.V., Grande J.P. (2002). IgA nephropathy. New Engl. J. Med..

[6] Mathieson P.W. (2003). Immune dysregulation in minimal change nephropathy. Nephrol. Dial. Transplant..

[7] Cameron J.S. (1999). Lupus nephritis. J. Am. Soc. Nephrol..

[8] Tian S.Y., Feldman B.M., Beyene J., Brown P.E., Uleryk E.M., Silverman E.D. (2014). Immunosuppressive therapies for the induction treatment of proliferative lupus nephritis: A systematic review and network metaanalysis. J. Rheumatol..

[9] Van Vollenhoven R.F., Mosca M., Bertsias G., Isenberg D., Kuhn A., Lerstrøm K., Aringer M., Bootsma H., Boumpas D., Bruce I.N. (2014). Treat-to-target in systemic lupus erythematosus: Recommendations from an international task force. Ann. Rheum. Dis..

[10] Ammon H., Anazodo M.I., Safayhi H., Dhawan B.N., Srimal R.C. (1992). Curcumin: A potent inhibitor of leukotriene B4 formation in rat peritoneal polymorphonuclear neutrophils (PMNL). Planta Medica..

[11] Jain S.K., Rains J., Jones K. (2006). Effect of curcumin on protein glycosylation, lipid peroxidation, and oxygen radical generation in human red blood cells exposed to high glucose levels. Free Radic. Biol. Med..

[12] Kowluru R.A., Kanwar M. (2007). Effects of curcumin on retinal oxidative stress and inflammation in diabetes. Nutr. Metab..

[13] Chainani-Wu N. (2003). Safety and anti-inflammatory activity of curcumin: A component of tumeric (Curcuma longa). J. Altern. Complementary Med..

[14] Cole G.M., Lim G.P., Yang F., Teter B., Begum A., Ma Q., Harris-White M.E., Frautschy S.A. (2005). Prevention of Alzheimer’s disease: Omega-3 fatty acid and phenolic anti-oxidant interventions. Neurobiol. Aging.

[15] Jagetia G.C., Aggarwal B.B. (2007). “Spicing up” of the immune system by curcumin. J. Clin. Immunol..

[16] Gao X., Kuo J., Jiang H., Deeb D., Liu Y., Divine G., Chapman R.A., Dulchavsky S.A., Gautam S.C. (2004). Immunomodulatory activity of curcumin: Suppression of lymphocyte proliferation, development of cell-mediated cytotoxicity, and cytokine production in vitro. Biochem. Pharmacol..

[17] Ammon H.P., Wahl M.A. (1991). Pharmacology of Curcuma longa. Planta Med..

[18] Mandal M.N., Patlolla J.M., Zheng L., Agbaga M.P., Tran J.T., Wicker L., Kasus-Jacobi A., Elliott M.H., Rao C.V., Anderson R.E. (2009). Curcumin protects retinal cells from light-and oxidant stress-induced cell death. Free Radic. Biol. Med..

[19] Jung K.A., Kwak M.K. (2010). The Nrf2 system as a potential target for the development of indirect antioxidants. Molecules.

[20] Wong S.H., Barlow J.L., Nabarro S., Fallon P.G., McKenzie A.N. (2010). Tim-1 is induced on germinal centre B cells through B-cell receptor signalling but is not essential for the germinal centre response. Immunology.

[21] Ichimura T., Brooks C.R., Bonventre J.V. (2012). Kim-1/Tim-1 and immune cells: Shifting sands. Kidney Int..

[22] Binion D.G., Heidemann J., Li M.S., Nelson V.M., Otterson M.F., Rafiee P. (2009). Vascular cell adhesion molecule-1 expression in human intestinal microvascular endothelial cells is regulated by PI 3-kinase/Akt/MAPK/NF-kappaB: Inhibitory role of curcumin. Am. J. Physiol..

[23] Banerjee M., Tripathi L.M., Srivastava V.M., Puri A., Shukla R. (2003). Modulation of inflammatory mediators by ibuprofen and curcumin treatment during chronic inflammation in rat. Immunopharmacol. Immunotoxicol..

[24] Deeb D., Jiang H., Gao X., Al-Holou S., Danyluk A.L., Dulchavsky S.A., Gautam S.C. (2007). Curcumin [1,7-bis(4-hydroxy-3-methoxyphenyl)-1-6-heptadine-3,5-dione; C21H20O6] sensitizes human prostate cancer cells to tumor necrosis factor-related apoptosis-inducing ligand/Apo2L-induced apoptosis by suppressing nuclear factor-kappaB via inhibition of the prosurvival Akt signaling pathway. J. Pharmacol. Exp. Ther..

[25] Guimarães M.R., Leite F.R.M., Spolidorio L.C., Kirkwood K.L., Rossa C. (2013). Curcumin abrogates LPS-induced pro-inflammatory cytokines in RAW 264.7 macrophages. Evidence for novel mechanisms involving SOCS-1,-3 and p38 MAPK. Arch. Oral Biol..

[26] Hanai H., Iida T., Takeuchi K., Watanabe F., Maruyama Y., Andoh A., Tsujikawa T., Fujiyama Y., Mitsuyama K., Sata M. (2006). Curcumin maintenance therapy for ulcerative colitis: Randomized, multicenter, double-blind, placebo-controlled trial. Clin. Gastroenterol. Hepatol..

[27] Hong J., Bose M., Ju J., Ryu J.H., Chen X., Sang S., Lee M.J., Yang C.S. (2004). Modulation of arachidonic acid metabolism by curcumin and related beta-diketone derivatives: Effects on cytosolic phospholipase A(2), cyclooxygenases and 5-lipoxygenase. Carcinogenesis.

[28] Lin J.K., Shih C.A. (1994). Inhibitory effect of curcumin on xanthine dehydrogenase/oxidase induced by phorbol-12-myristate-13-acetate in NIH3T3 cells. Carcinogenesis.

[29] Ghoneim A.I., Abdel-Naim A.B., Khalifa A.E., El-Denshary E.S. (2002). Protective effects of curcumin against ischaemia/reperfusion insult in rat forebrain. Pharmacol. Res..

[30] Manikandan R., Thiagarajan R., Beulaja S., Sudhandiran G., Arumugam M. (2010). Curcumin protects against hepatic and renal injuries mediated by inducible nitric oxide synthase during selenium-induced toxicity in Wistar rats. Microsc. Res. Tech..

[31] Chainani-Wu N., Silverman S., Reingold A., Bostrom A., Mc Culloch C., Lozada-Nur F., Weintraub J. (2007). A randomized, placebo-controlled, double-blind clinical trial of curcuminoids in oral lichen planus. Phytomedicine.

[32] Baum L., Lam C.W., Cheung S.K., Kwok T., Lui V., Tsoh J., Lam L., Leung V., Hui E., Ng C. (2008). Six-month randomized, placebo-controlled, double-blind, pilot clinical trial of curcumin in patients with Alzheimer disease. J. Clin. Psychopharmacol..

[33] Usharani P., Mateen A.A., Naidu M.U., Raju Y.S., Chandra N. (2008). Effect of NCB-02, atorvastatin and placebo on endothelial function, oxidative stress and inflammatory markers in patients with type 2 diabetes mellitus: A randomized, parallel-group, placebo-controlled, 8-week study. Drugs R D.

[34] Khajehdehi P., Pakfetrat M., Javidnia K., Azad F., Malekmakan L., Nasab M.H., Dehghanzadeh G. (2011). Oral supplementation of turmeric attenuates proteinuria, transforming growth factor-beta and interleukin-8 levels in patients with overt type 2 diabetic nephropathy: A randomized, double-blind and placebo-controlled study. Scand. J. Urol. Nephrol..

[35] Agarwal K.A., Tripathi C.D., Agarwal B.B., Saluja S. (2011). Efficacy of turmeric (curcumin) in pain and postoperative fatigue after laparoscopic cholecystectomy: A double-blind, randomized placebo-controlled study. Surg. Endosc..

[36] Golombick T., Diamond T.H., Manoharan A., Ramakrishna R. (2012). Monoclonal gammopathy of undetermined significance, smoldering multiple myeloma, and curcumin: A randomized, double-blind placebo-controlled cross-over 4g study and an open-label 8g extension study. Am. J. Hematol..

[37] Wu T., Qin X., Kurepa Z., Kumar K.R., Liu K., Kanta H., Zhou X.J., Satterthwaite A.B., Davis L.S., Mohan C. (2007). Shared signaling networks active in B cells isolated from genetically distinct mouse models of lupus. J. Clin. Investigig..

[38] Wu T., Fu Y., Brekken D., Yan M., Zhou X.J., Vanarsa K., Deljavan N., Ahn C., Putterman C., Mohan C. (2010). Urine proteome scans uncover total urinary protease, prostaglandin D synthase, serum amyloid P, and superoxide dismutase as potential markers of lupus nephritis. J. Immunol..

[39] Xie C., Sharma R., Wang H., Zhou X.J., Mohan C. (2004). Strain distribution pattern of susceptibility to immune-mediated nephritis. J. Immunol..

[40] Wu T., Ye Y., Min S.Y., Zhu J., Khobahy E., Zhou J., Yan M., Hemachandran S., Pathak S., Zhou X.J. (2014). Prevention of murine lupus nephritis by targeting multiple signaling axes and oxidative stress using a synthetic triterpenoid. Arthritis Rheumatol..

[41] Carlsson A., Wuttge D.M., Ingvarsson J., Bengtsson A.A., Sturfelt G., Borrebaeck C.A., Wingren C. (2011). Serum protein profiling of systemic lupus erythematosus and systemic sclerosis using recombinant antibody microarrays. Mol. Cell. Proteom..

[42] Chandran B., Goel A. (2012). A randomized, pilot study to assess the efficacy and safety of curcumin in patients with active rheumatoid arthritis. Phytother. Res..

[43] Aziz M.T.A., El-Asmar M.F., El-Ibrashy I.N., Rezq A.M., Al-Malki A.L., Wassef M.A., Fouad H.H., Ahmed H.H., Taha F.M., Hassouna A.A. (2012). Effect of novel water soluble curcumin derivative on experimental type-1 diabetes mellitus (short term study). Diabetol. Metab. Syndr..

[44] Sun J., Zhao Y., Hu J. (2013). Curcumin inhibits imiquimod-induced psoriasis-like inflammation by inhibiting IL-1beta and IL-6 production in mice. PloS ONE.

[45] Ung V.Y., Foshaug R.R., MacFarlane S.M., Churchill T.A., Doyle J.S., Sydora B.C., Fedorak R.N. (2010). Oral administration of curcumin emulsified in carboxymethyl cellulose has a potent anti-inflammatory effect in the IL-10 gene-deficient mouse model of IBD. Dig. Dis. Sci..

[46] Ireson C., Orr S., Jones D.J., Verschoyle R., Lim C.-K., Luo J.-L., Howells L., Plummer S., Jukes R., Williams M. (2001). Characterization of metabolites of the chemopreventive agent curcumin in human and rat hepatocytes and in the rat in vivo, and evaluation of their ability to inhibit phorbol ester-induced prostaglandin E2 production. Cancer Res..

[47] Forward N.A., Conrad D.M., Coombs M.R.P., Doucette C.D., Furlong S.J., Lin T.-J., Hoskin D.W. (2011). Curcumin blocks interleukin (IL)-2 signaling in T-lymphocytes by inhibiting IL-2 synthesis, CD25 expression, and IL-2 receptor signaling. Biochem. Biophys. Res. Commun..

[48] Kliem C., Merling A., Giaisi M., Köhler R., Krammer P.H., Li-Weber M. (2012). Curcumin suppresses T cell activation by blocking Ca2+ mobilization and nuclear factor of activated T cells (NFAT) activation. J. Biol. Chem..

[49] Han S.-S., Chung S.-T., Robertson D.A., Ranjan D., Bondada S. (1999). Curcumin causes the growth arrest and apoptosis of B cell lymphoma by downregulation of egr-1, c-myc, bcl-X L, NF-κB, and p53. Clin. Immunol..

[50] Ghosh A.K., Kay N.E., Secreto C.R., Shanafelt T.D. (2009). Curcumin inhibits prosurvival pathways in chronic lymphocytic leukemia B cells and may overcome their stromal protection in combination with EGCG. Clin. Cancer Res..

[51] Brouet I., Ohshima H. (1995). Curcumin, an anti-tumor promoter and anti-inflammatory agent, inhibits induction of nitric oxide synthase in activated macrophages. Biochem. Biophys. Res. Commun..

[52] Young N.A., Bruss M.S., Gardner M., Willis W.L., Mo X., Valiente G.R., Cao Y., Liu Z., Jarjour W.N., Wu L.-C. (2014). Oral administration of nano-emulsion curcumin in mice suppresses inflammatory-induced NFκB signaling and macrophage migration. PloS ONE.

[53] Liu T., Li C., Sun H., Luo T., Tan Y., Tian D., Guo Z. (2014). Curcumin inhibits monocyte chemoattractant protein-1 expression and enhances cholesterol efflux by suppressing the c-Jun N-terminal kinase pathway in macrophage. Inflamm. Res..

[54] Castro C., Barcala Tabarrozzi A., Winnewisser J., Gimeno M., Antunica Noguerol M., Liberman A., Paz D., Dewey R., Perone M. (2014). Curcumin ameliorates autoimmune diabetes. Evidence in accelerated murine models of type 1 diabetes. Clin. Exp. Immunol..

[55] Rogers N., Kireta S., Coates P. (2010). Curcumin induces maturation-arrested dendritic cells that expand regulatory T cells in vitro and in vivo. Clin. Exp. Immunol..

[56] Bright J.J. (2007). Curcumin and autoimmune disease. The Molecular Targets and Therapeutic Uses of Curcumin in Health and Disease.

[57] Kang D., Li B., Luo L., Jiang W., Lu Q., Rong M., Lai R. (2016). Curcumin shows excellent therapeutic effect on psoriasis in mouse model. Biochimie.

[58] Khajehdehi P., Zanjaninejad B., Aflaki E., Nazarinia M., Azad F., Malekmakan L., Dehghanzadeh G.-R. (2012). Oral supplementation of turmeric decreases proteinuria, hematuria, and systolic blood pressure in patients suffering from relapsing or refractory lupus nephritis: A randomized and placebo-controlled study. J. Ren. Nutr..

